# Medical Extracts

**Published:** 1884-12

**Authors:** 


### Medical Extracts.

---

### Muriate of Cocaine, a new local Anæsthetic.

Most remarkable effects are ascribed to this drug, the invention of Dr. Koller, of Vienna. As yet it has had but a limited trial in England, but in America and in Germany its use, in eye operations at least, has now become quite general. There seems to be no doubt as to its extraordinary power of rendering mucous membranes insensible, if we are to judge from the already abundant clinical records in the American and German journals.

We append Messrs. Allen and Hanbury's account of the drug:—

"This salt of cocaine (the alkaloidal principle derived from the leaves of the coca plant—*Erythroxylon Coca*) has lately come into prominent use as a local anæsthetic for delicate mucous surfaces. Whilst it has been successfully tested on the different mucous surfaces of the ear, nose, mouth, urethra, vagina, and rectum, it has been principally used as a means of causing anæsthesia of the eye, and in such application has been successfully used by operators both in Germany and the United States, and by several surgeons in this country. For this especial purpose its use and success were shown in several operations performed at the Ophthalmic Congress recently held in Heidelberg.

"Numerous cases show that the application of a few drops of a 2°/₀, or, better, of a 4°/₀ solution of muriate of cocaine to the eye causes, within about fifteen minutes from the first application, anæsthesia of the conjunctiva, cornea and surroundings, an anæsthesia so complete that operations for cataract, squint, &c., can be performed during its influence without giving pain to the patient, and without causing any subsequent injury or discomfort to the organ beyond a transient dilatation of the pupil, which is not accompanied by any material interference with sight, and which passes away in a brief time. As previously mentioned, the action of the muriate of cocaine is similar upon the mucous surfaces of other organs, and gives rise to no unpleasant symptoms. It will thus be seen that in this preparation we possess an invaluable agent for producing a safe and satisfactory local anæsthesia of parts intensely sensitive.

"The coca leaves yield only a very minute percentage of the alkaloid, consequently the cost of the material is considerable; but, as the quantity required to produce anæsthesia is very small, its cost becomes a trifling matter compared with the immense benefits derivable from its use.

"The mode of procedure recommended in operations on the eye is as follows:—

"A 2°/₀ solution of muriate of cocaine in water is used. Two or three drops are instilled in the usual way on the lower lid. Five minutes afterwards the application is repeated, and after five minutes more the same quantity is again applied. As a rule the anæsthesia is complete at the end of fifteen minutes (often less) from the time of first instillation, and the cornea, and all the surroundings of the eye, may be manipulated at will without causing the slightest pain. The anæthesia generally remains for a period varying from twenty minutes to half an hour, and passes away without causing any discomfort. The pupil becomes dilated about six or eight minutes after the commencement of complete anæsthesia.

"Experience has shown that a 4°/₀ solution is much more effective and reliable.

"Cocaine (and its salts) has likewise been strongly recommended for internal use, in small doses, as a powerful nervous stimulant, in full doses as a sedative; and, in the experience of some, has had marked value in preventing craving for alcohol and morphia. Indeed, it has been asserted that the use of morphia may be suddenly stopped without danger to the patient, and without causing the desire for it, if cocaine be carefully administered. On these points, however, further testimony is required to ensure confidence in the use of cocaine."

### The Oleate of Copper in Parasitic Skin Diseases.

The recognition of the fact of parasites as the cause of a very large proportion of cutaneous diseases marked a very decided advance in the therapeutics of these affections. The fact having been recognized, the only question which presented itself was the discovery of an agent which should destroy the parasite without causing in the skin itself a greater disturbance than that caused by the insect. It required a number of years for this discovery. Many were the drugs recommended to this end; but the crucial tests of experience showed them nearly all to be indifferently adapted to the end for which they were designed. Possibly, the fact of the ignorance of such a solvent as would most effectually carry the parasiticide to the exact location of the parasite, and thus insure its actual contact therewith, may have been largely to blame. If this was the case, the desideratum has been very fully supplied in oleic acid. Certainly the salts prepared with this acid, which have been introduced to the profession within a comparatively recent time, have placed in the hands of the profession more effectual means than had been previously enjoyed for the treatment of skin affections of a parasitic nature.

In an article in the August 30th number of the *New York Medical Journal*, Dr. F. Le Sieur Weir, clinical professor of diseases of the skin in the Medico-Chirurgical College of Philadelphia, gives a comprehensive report of the results of his use of the oleate of copper in five hundred cases of parasitic disease of the skin. He prefaces this report with some remarks, with which we are not particularly interested at this time, on the manner of preparing this salt so that its most beneficial action may be secured. The oleate of copper, he remarks, as well as all other oleates, prepared after the manner described, is, to all intents and purposes, a saturated solution; and he regards the existence of any free oleic acid in the preparation as an error which is very apt to complicate the results of the employment of the oleate proper.

Dr. Weir claims to have had exceptional opportunities for testing the curative action of the oleate of copper in parasitic diseases of the skin, and his cases cover no less than seven different forms of such disease; namely: Tinea tonsurans, T. circinata, T. kerion, T. sycosis, T. versicolor, T. favosa and eczema marginatum. In his employment of the various remedies in vogue before the introduction of the oleate of copper, he found that the remedies diminished in their therapeutic activity with the length of time during which they were employed. This objection does not apply to the oleate of copper. His plan of treatment is as follows: From hairy parts he first cuts off the hair close to the skin, over the immediate part affected, and clips an area extending at least one inch beyond the affected surface. The parts are then anointed with fluid cosmoline, or glycerine, or a bread-and-milk poultice is applied, for the purpose of dislodging such scales, crusts, scurf, or actual dirt, as may have accumulated. The ointment of the oleate of copper, of such strength as is suited to the severity of the case, is then rubbed into the diseased patches gently, but thoroughly, so as to secure as complete and rapid absorption as possible. The parts should then be covered or left bare, as the exigencies of the case may dictate. The process of inunction should be repeated at least twice daily, this being usually amply sufficient. The strength of the ointment varies with the severity of the case, from one drachm to six of the cupric oleate, to the ounce of cosmoline. The results of this treatment, as reported, are certainly very remarkable, few cases requiring its continuance for over two weeks.

It is interesting to note that Dr. Weir regards epilation as a quite unnecessary procedure in the treatment of the diseases above referred to. If additional experience shall show that the use of this preparation may obviate the necessity of epilation, the value of the treatment must be very greatly enhanced. In barber's itch, epilation has heretofore been regarded as an essential step in the treatment; but Dr. Weir emphasizes the statement that he has rarely found it necessary under the use of the oleate of copper, even in this affection. When the disease attacks the eyelashes it is more or less necessary, inasmuch as without it a satisfactory application of the ointment cannot be made.—*The Therapeutic Gazette*, October, 1884.

### Operative Opening of Pulmonary Cavities.

Before the late International Medical Congress, Dr. E. Bull (Christiania), in a paper on this subject, laid down the following propositions:—

1. Abscesses of the lung, which can be diagnosed with certainty, and are so situated that they can be opened through the chest-wall, should be treated in the same way as pleural empyema.

2. The condition is the same with regard to limited gangrene of the lung. If several gangrenous foci exist, each one must be treated separately.

3. Echinococci, and 4, foreign bodies in the lung, are to be treated in a similar manner.

5. In bronchiectasis the formation of a pulmonary fistula is indicated only when the accumulation of stagnant matter in large cavities essentially contributes to the deterioration of the patient's condition.

6. In rare cases of tuberculosis, where a large cavity is the predominating condition, the cavity may be laid open with the view of improving the condition of the patient.

7. The operative puncture of a pulmonary fistula is justifiable as a palliative measure.

8. In cases where diagnosis cannot be arrived at, exploratory puncture is certainly of much value; positive as well as negative results may be derived from it.

9. Adhesion of the layers of the pleura, though not to be insisted on as an absolutely necessary preliminary to the opening of pulmonary cavities.

10. Amyloid degeneration is not an absolute contra-indication to a palliative operation.

11. The use of the thermo-cautery is to be recommended both for the opening of cavities and for the destruction of diseased portions of lung-tissue.—*Medical and Surgical Reporter*, Oct. 11th, 1884.

**On the action of Antipyrin.**

Dr. Sassjezky has used antipyrin in three cases in doses of 1 to 2 grammes, and comes to the following conclusions:—

1. That it has great temperature reducing power, a temperature of 41° C. in the rectum being brought down to normal by it. He found that one hour after the administration of 2 grammes a fall of from .4 to 1·1° C. was noted, but that this depression reached the maximum three or four hours later, and continued from three to seven hours.

2. Profuse perspiration, reminding one of the action of pilocarpine, comes on two or three hours after the drug is taken in the above dose.

3. The pulse an hour after its administration was slower, after having been to a small extent quickened. It never becomes weaker; on the contrary, somewhat stronger.

4. The temperature of the periphery of the body falls to an extent proportionate to that of the rectum. There were no disagreeable accompaniments observed.

Dr. A. Cahn says that from cases observed in Professor Kussmaul's practice, where antipyrin was used in acute febrile affections, such as typhoid, croupous and catarrhal pneumonia, erysipelas faciei, and pleurisy, depression of temperature always followed its administration, and when the temperature rose again rigor was never seen. In several cases of pneumonia, after large doses of the drug had been taken on the fifth or sixth day of the disease, the temperature remained sub-febrile for some days, although the character of the pulse and respiration showed that the crisis had not taken place. This observation shows that antipyrin not only acts against fever as a symptom, but that it is capable of influencing directly the pathological process. The "antipyrin erythema" which had been previously seen in the cliniques of Breslau and Zürich, was also observed in Strassburg in several cases of typhoid. The erythematory eruption makes its appearance without any subjective symptom, and without any influence on the course of the fever, and consists of round, brownish-red spots, somewhat raised, and which disappear entirely under the pressure of the finger. The extensor surfaces had more spots on them than the flexor, the back than the chest or abdomen, and the head, palms of the hands, and soles of the feet remained quite free from it. After the administration of the drug was stopped the rash got paler, but reappeared when its use was resumed.—*Deutsche Mediz. Zeitung*, Oct. 27th, 1884.

**Tetanus following hypodermic injection of Quinine.**

Dr. Bartolozzi, in a communication to the *Gazz. degli Ospitali*, says that he has observed tetanus which terminated fatally follow the hypodermic injection of quinine in two cases out of about two thousand injections, where the drug was thus used during the years 1878 to 1881 in a malarial district. The first case was that of a woman who, whilst showing symptoms of marsh fever, was attacked by severe pleuro-pneumonia; two injections were administered daily for two days—the first into the arm, the second into the thigh—each consisting of—

| | | |
|---|---|---|
| Morph. Muriat. ... ... | ·01 | gramme. |
| Quinine Sulph. ... ... | ·30 | " |
| Aquam ad. ... ... ... | 2·00 | " |

On the thigh a small abscess formed, and the symptoms of tetanus made their appearance after five days.

In the second case, tetanus followed hypodermic injection of quinine, which was administered on account of an attack of malarial fever, in a person suffering from hystero-epilepsy.

Both cases occurred in the spring, with only a short interval between them, at a time when rheumatic affections were prevalent. The author of the paper thinks that some peculiar atmospheric conditions, combined with the pronounced neuropathic character of the patients, taken together, were the predisposing causes of the outbreak of tetanus, which was observed to follow the irritation of the quinine injection.

Dr. Rossi relates another case of a young man, 18 years old, of lymphatic temperament, who came under treatment in November, 1881, with symptoms of pulmonary tuberculosis. In March, 1882, the patient was able to do his daily work again, but in October of that year he became worse; and his temperature being high, and quinine given by the mouth only producing a temporary effect, hydrochlorate of quinine was used hypodermically for some time, and entirely controlled the febrile symptoms. After every injection symptoms of slight irritation manifested themselves, consisting of swelling, circumscribed redness, and pain on pressure, which disappeared after a day or two. In March, 1883, the febrile condition again coming on, the patient wished to have the injections resumed, and for three consecutive days two injections were introduced into the upper and lower arm of each side alternately each day (the hydrochlorate, of the same quality and in the same quantity, being always used). After the last injection symptoms of more severe irritation appeared, the pain of which, however, cataplasmata quickly controlled. Four days after the last injection the pain became worse, and redness and swelling appeared at the site of puncture; suppuration did not result from this, and under suitable treatment the inflammation gradually subsided. On the 29th March the patient felt weak to an extreme degree, and when sitting up in bed he could not keep his head up. On the same day trismus appeared; on the next day pronounced tetanus came on, and during the following night, about thirty hours after the first appearance of symptoms of trismus, the patient died.—*Deutsche Mediz. Zeitung*, Nov. 6th, 1844.

**Jequirity.**

Dr. S. Pollak, of St. Louis, thus summarises his extensive experience of jequirity:—

1. Jequirity is one of the most interesting and surprising therapeutic agents; very potent, yet limited in its effect.

2. It is the most reliable and prompt remedy in the treatment of trachoma and pannus.

3. The more inveterate the granulations, the more efficient and striking the result.

4. Adults are more amenable to treatment than young individuals.

5. Abrasions, or even ulcerations of the cornea are no bar to its cautious use.

6. Pyorrhœa is no essential factor in the treatment, but a sero-purulent discharge is, as well as the formation of a membrane on the palpebral conjunctiva.

7. Atrophy of the conjunctiva and formation of cicatricial tissue may be exceptional occurrences; they have never been met with by me.

8. A sound bean, and a fresh infusion—not more than 48 hours old, is a *conditio sine qua non;* the deterioration of the infusion is so rapid, that in three days it swarms with bacteria, becomes putrid, fetid and irritating, and unfit for use.

9. The maximum strength of the infusion need not exceed 3 per cent., and a maceration of a few hours suffices to abstract all its medicinal properties. A stronger infusion, and a too frequent application, may induce destructive purulency.

10. Opiates can be dispensed with, for all painful symptoms subside on the discontinuance of the jequirity.—*Amer. Journ. of Ophthalmology*, June 15th, 1884.

### Massage.

Dr. Louis Henry, in the *Australian Medical Journal*, Aug. 15th, 1884, gives the following practical instructions for the use of massage:—

1. The most ordinary movement in the carrying out of this treatment is the centripetal. This means the direction of all the strokes towards the centre of the body, or to the heart. The stroking of the legs to be upwards, and of the head and neck downwards, in fact in that direction in which veins and lymphatics are unloaded. The hands of the operator should be anointed with lard, oil, or vaseline, and the palms of the hands must be soft and fleshy, for horny and rough hands are a cause of irritation. The hair on the skin of the patient should be removed by shaving, or cut short with scissors, as constant rubbing and friction very soon sets up an inflammatory condition of the hair bulbs, which causes the patient pain, and compels the operator to desist until the angry appearances have subsided. After each sitting the parts operated on must be enveloped in a bandage or roller of flannel, so as to retain the heat and continue the nervous reaction as long as possible.

Application No. 2 is *massage à friction*. A different movement is made with each hand, the object being to disseminate with one hand what the other forces towards the centre. It is a kind of milking movement. The one hand rubs across and across in a sort of circular movement, while the other makes centripetal parallel strokes. It is a difficult movement, which requires a good deal of practice.

No. 3 is *petrisage*, from *petrir*, to knead. Here you squeeze between the fingers and thumb, raising up the muscles and soft parts—an excellent movement for indurated inflammatory products or hæmorrhagic exudations.

No. 4 is *tapotement*, from *taper*, to strike. Generally the ulnar side of the first and second fingers, or the margin of the hand is used, and applied like the percussion hammer in quick and sharp successive strokes, frequently applied for neuralgic and rheumatic pains, hyperæsthetic and anæsthetic symptoms, and paralysis. The other auxiliary manipulations are active and passive muscular action, electro therapeutics, compression with bandages, baths, forcible rupture of adhesions, &c. After massaging a part we proceed to the passive movement, which consists in the movement of the limb by the masseur, and not till after a prolonged period of treatment will it be found advisable to allow the patient to indulge in independent movements, which are called active. The action of a muscle is like that of a pump, for those masses or fluids thrown into motion by massage are carried further on by the muscular action. The length of a sitting must depend on the nature of the complaint and the character of the treatment. Sometimes twice daily, from six to ten minutes; sometimes, as in nervous disorders, from half an hour to one hour.

### Germ Theory and Heroic Doses.

Any one who has been a close observer must have noticed the growth of therapeutic skepticism in certain quarters during the past couple of decades. This growth has been most rapid among those who have had really the least experience in the treatment of disease, their studies and observations being rather in the line of physiological and pathological research. While the active, working practitioner has been but very slightly affected by these views sought to be promulgated by these authors, it is to be feared that the young practitioners are inclined to carry with them into the active duties of their profession the influence of the teachings of those under whom their medical knowledge was acquired. It is the opinion of the venerable Editor of the *Journal* of the American Medical Association that there is setting in a reaction from this skepticism and expectancy in the treatment of disease, and that the cause of this reaction is largely traceable to the rapid development of the doctrine of germ etiology, and consequent germicide therapeutics. This reaction began with the revival of the antipyretic treatment of fevers, and it is already so great as to justify the fear that patients may be placed in greater danger from the large doses of microbe remedies administered in some cases than from the microbes themselves. Our contemporary very judiciously cautions against practitioners going to too great lengths in this direction. He thinks that the large doses of corrosive sublimate which have latterly been recommended are extremely dangerous, and he advises practitioners against a too ready acceptance of the rose-coloured opinions expressed of the action of this germicide. While, doubtless, there are numerous affections in which the destruction of the bacteria is a consummation devoutly to be wished, the fact should never be lost sight of that there is a danger, while pursuing the parasite, of destroying the patient whom it infects. We would heartily coincide with our contemporary, and admonish our readers that they be not carried away by the weight of authority under which the use of the heroic doses of the bi-chloride of mercury is becoming so popular.—*Therapeutic Gazette*, September, 1884.

